# Integrating predictive coding and a user-centric interface for enhanced auditing and quality in cancer registry data

**DOI:** 10.1016/j.csbj.2024.04.007

**Published:** 2024-04-07

**Authors:** Hong-Jie Dai, Chien-Chang Chen, Tatheer Hussain Mir, Ting-Yu Wang, Chen-Kai Wang, Ya-Chen Chang, Shu-Jung Yu, Yi-Wen Shen, Cheng-Jiun Huang, Chia-Hsuan Tsai, Ching-Yun Wang, Hsiao-Jou Chen, Pei-Shan Weng, You-Xiang Lin, Sheng-Wei Chen, Ming-Ju Tsai, Shian-Fei Juang, Su-Ying Wu, Wen-Tsung Tsai, Ming-Yii Huang, Chih-Jen Huang, Chih-Jen Yang, Ping-Zun Liu, Chiao-Wen Huang, Chi-Yen Huang, William Yu Chung Wang, Inn-Wen Chong, Yi-Hsin Yang

**Affiliations:** a Intelligent System Laboratory, Department of Electrical Engineering, College of Electrical Engineering and Computer Science, National Kaohsiung University of Science and Technology, Kaohsiung 80778, Taiwan; b National Institute of Cancer Research, National Health Research Institutes, Tainan 70456, Taiwan; c School of Post-Baccalaureate Medicine, College of Medicine, Kaohsiung Medical University, Kaohsiung 80708, Taiwan; d Center for Big Data Research, Kaohsiung Medical University, Kaohsiung 80708, Taiwan; e Electromagnetic Sensing Control and AI Computing System Laboratory, Department of Electrical Engineering, College of Electrical Engineering and Computer Science, National Kaohsiung University of Science and Technology, Kaohsiung 80778, Taiwan; f Department of Computer Science, National Yang Ming Chiao Tung University, Hsinchu, Taiwan, ROC; g Advanced Technology Laboratory, Chunghwa Telecom Laboratories, Taoyuan, Taiwan, ROC; h Cancer Center, Kaohsiung Medical University Hospital, Kaohsiung 80708, Taiwan; i Division of Pulmonary and Critical Care Medicine, Kaohsiung Medical University Hospital, Kaohsiung Medical University, Kaohsiung 80708, Taiwan; j Department of Medical Information, Kaohsiung Medical University Hospital, Kaohsiung 80708, Taiwan; k Department of Radiation Oncology, Kaohsiung Medical University Hospital, Kaohsiung Medical University, Kaohsiung 80708, Taiwan; l Health Promotion Administration, Ministry of Health and Welfare, Taipei 10341, Taiwan; m Waikato Management School, University of Waikato, Hamilton, New Zealand; n Division of Chest Medicine, Kaohsiung Medical University Hospital, Kaohsiung Medical University, Kaohsiung 80708, Taiwan; o Department of Biological Science and Technology, National Yang Ming Chiao Tung University, Hsinchu 30010, Taiwan

**Keywords:** Natural language processing, Cancer registry, Electronic health record, Patient journey

## Abstract

Data curation for a hospital-based cancer registry heavily relies on the labor-intensive manual abstraction process by cancer registrars to identify cancer-related information from free-text electronic health records. To streamline this process, a natural language processing system incorporating a hybrid of deep learning-based and rule-based approaches for identifying lung cancer registry-related concepts, along with a symbolic expert system that generates registry coding based on weighted rules, was developed. The system is integrated with the hospital information system at a medical center to provide cancer registrars with a patient journey visualization platform. The embedded system offers a comprehensive view of patient reports annotated with significant registry concepts to facilitate the manual coding process and elevate overall quality. Extensive evaluations, including comparisons with state-of-the-art methods, were conducted using a lung cancer dataset comprising 1428 patients from the medical center. The experimental results illustrate the effectiveness of the developed system, consistently achieving F1-scores of 0.85 and 1.00 across 30 coding items. Registrar feedback highlights the system’s reliability as a tool for assisting and auditing the abstraction. By presenting key registry items along the timeline of a patient’s reports with accurate code predictions, the system improves the quality of registrar outcomes and reduces the labor resources and time required for data abstraction. Our study highlights advancements in cancer registry coding practices, demonstrating that the proposed hybrid weighted neural-symbolic cancer registry system is reliable and efficient for assisting cancer registrars in the coding workflow and contributing to clinical outcomes.

## Graphical Abstract

**Figure:**
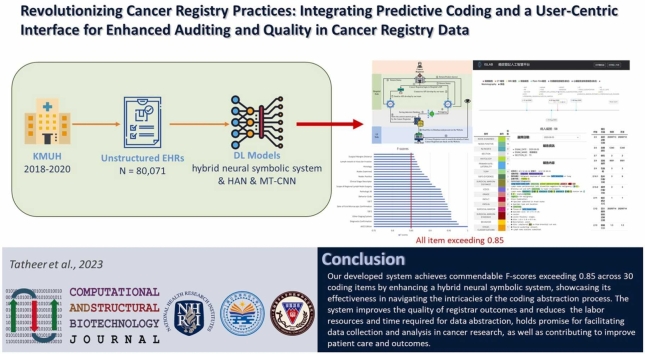

## 1 Introduction

In 2020, lung cancer was the most fatal form of cancer, causing approximately 1.8 million deaths [1], according to the Global Cancer Observatory, an initiative established by the International Agency for Research on Cancer. The United States is estimated to witness 1918,030 new cancer cases and 609,360 cancer-related deaths in 2022. Among these, 350 deaths per day would be attributed to lung cancer, which remains the primary cause of cancer mortality [2]. Similarly, for years, lung cancer has been Taiwan’s leading cause of cancer-specific deaths. Therefore, implementing effective cancer control measures is crucial to prevent cancer and ensure affordable and accessible treatment and care for individuals [3].

Cancer registries play a critical role in cancer control because they provide essential data for surveillance, research, planning, quality improvement, and evaluation. They contribute to the understanding of cancer trends, monitor the performance of cancer control programs, support evidence-based decision-making, and facilitate targeted interventions to reduce the burden of cancer in populations [4]. Taiwan’s Cancer Registry Center (TCRC) has maintained the dataset of the official cancer registry since 1979. Although cancer registries contain valuable data, the delay in reporting hinders their use for generating real-time, actionable outcome and quality reports.

According to the research conducted by Jabour, Dixon [5] the cancer registry process involves three primary steps: case finding, abstraction, and reporting. Among these steps, abstraction is the most time-consuming step, requiring approximately 45 min to 1.5 h per case on a daily basis [5]. According to our study at Kaohsiung Medical University Chung-Ho Memorial Hospital (KMUH) in Taiwan, the estimated time for processing one case is approximately 30 min. The abstraction process involves the extraction of tumor-related information, as well as information about staging, diagnostic studies, and treatment from various sections within the medical record. A notable challenge that contributes to the time-intensive nature of this process is the sheer volume and diversity of reports associated with each patient. Registrars are expected to review and comprehend a multitude of medical reports, including but not limited to pathology reports, radiology reports, and discharge summaries, which often span over a period of approximately 1.5 years per patient. To illustrate the extent of information processing involved, consider that, on average, each patient's medical record contains 4.8 pathology reports and a staggering 47.6 image reports based on our collected data. The statistics figures underscore the substantial amount of data that necessitates careful review and extraction. In pursuit of expediting the curation process while maintaining a high standard of quality, we describe work that we have conducted to develop a lung cancer registry coding system. This system harnesses the power of natural language processing (NLP) to autonomously retrieve clinical data elements pertaining to lung cancer from unstructured clinical text reports encompassing a patient’s medical journey.

### 1.1 Prior work

Table 1 provides a summary of the extant literature conducted on the automated extraction of cancer-related data. One notable open-source system, MedTAS/P, was introduced in 2009 by Coden, Savova [6]. The system uses a combination of machine learning and hand-craft matching rules to extract cancer registry-related information such as histology, grade, tumor size, and lymph node attributes from free-text pathology reports. Kavuluru, Hands [7] proposed to extract unigrams, bigrams, and named entities as features for three machine learning algorithms, including logistic regression, naïve Bayes, and support vector machines (SVMs) to extract 57 generic sites of ICD-O-3 (international classification of diseases for oncology, 3rd edition) codes from pathology reports. However, the traditional *n*-gram feature representation of free text documents often fails to capture word ordering and semantics, thus compromising text comprehension. To overcome the limitations, Yoon, Roberts [8] developed a graph-of-words text representation based on graph analytics for automated extraction of histologic grade from pathology reports.

**Table 1:** Comparison of methods proposed for the extraction of cancer-related information.

Research	Approach			Strategy			Task		Cancer		Source
	R	ML	DL	STL	MTL	H	SIE	MIE	S	M	
Coden, Savova[6]	✓	✓		✓				✓	✓		PRs
Kavuluru, Hands[7]		✓		✓			✓			✓	PRs
Yoon, Ramanathan[13]		✓	✓	✓	✓			✓		✓	PRs
Yoon, Roberts[8]		✓		✓			✓			✓	PRs
Qiu, Yoon[9]		✓	✓	✓			✓			✓	PRs
Gao, Young[11]		✓	✓	✓				✓		✓	PRs
Alawad, Yoon[14]		✓	✓	✓	✓			✓		✓	PRs
Yoon, Gounley[15]			✓		✓			✓		✓	PRs
Dubey, Hinkle[10]		✓	✓	✓			✓			✓	PRs
Yoon, Gounley[16]			✓		✓			✓		✓	PRs
Alawad, Gao[17]		✓	✓	✓	✓			✓		✓	PRs
Dai, Yang[12]			✓			✓		✓	✓		PRs
Yoon, Stanley[18]			✓		✓			✓		✓	PRs
This Study	✓	✓	✓			✓		✓	✓		PRs, IRs

Abbreviations. R: Rule-based, ML: Machine Learning, DL: Deep Learning, STL: Single-task Learning, MTL: Multi-task Learning, H: Hybrid, SIE: Single Information Extraction, MIE: Multiple Information Extraction, S: Single, M: Multiple, PRs: Pathology Reports, IRs: Image Reports

Recent advancements in deep neural networks have showcased their remarkable ability to extract information with superior performance, comparing to conventional classification techniques. Qiu, Yoon [9] investigated the application of a convolutional neural network (CNN) for extracting ICD-O-3 topographic codes from breast and lung cancer pathology reports. They compared the performance of the developed CNN with a conventional term frequency-inverse document frequency (TF-IDF) approach. Their results demonstrate the potential of applying deep learning-based approach for the automated abstraction of pathology reports. Dubey, Hinkle [10] proposed to combine deep learning techniques with a *k*-nearest neighbors classifier to extract tumor site information from pathology reports. They used the localized sliced inverse-regression method to learn a low-dimensional representation to improve the accuracy of tumor site extraction even with high dimensional data and limited labeled samples. A noteworthy example of utilizing deep learning methods for the extraction of cancer information is the work presented by Gao, Young [11], who applied the hierarchical attention network (HAN) to extract information from pathology reports. They demonstrated that HAN performed signiﬁcantly better compared to the conventional machine learning and neural network-based techniques. In particular, they highlighted the pre-training process based on TF-IDF-weighted word embeddings can improve the performance in the primary site classification task. Another study presented by Dai, Yang [12] proposed a hybrid neural symbolic method for cancer registry coding. Their research primarily focused on the challenge of transfer learning across different hospitals. The authors demonstrated the proposed hybrid method exhibited greater robustness compared to both HAN and BERT-based models.

Recent studies have expressed interest in employing multi-task learning to address the coding of registry data elements with shared information characteristics during the abstraction task in the cancer registry process. Yoon, Ramanathan [13] investigated the development of two deep feed-forward neural networks configured with the sharing of the same input and hidden neurons for automated extraction of the primary cancer site and its laterality. Their results indicate the strong potential of multi-task neural networks for the cancer registry coding task. Later Alawad, Yoon [14] and Alawad, Gao [17] developed a multi-task CNN (MT-CNN) model for extracting the primary site, histological grade, and laterality from pathology reports and demonstrated the performance of MT-CNN consistently outperformed single-task CNNs and SVMs. Yoon, Gounley [15] explored the application of graph-based CNNs trained with graph-of-word representation in a manner of multi-task learning for four coding tasks namely sub-site, laterality, behavior, and grade, and illustrated the superior performance of their method compared to other approaches. Yoon, Gounley [16] proposed to apply Bayesian optimization method to find the optimized hyper-parameters for MT-CNN and hierarchical convolutional attention network on a synthesized dataset based on de-identified pathology reports for the coding tasks of site, laterality, behavior and grade. They demonstrated that Bayesian optimization is a feasible approach to optimize the performance of these models.

Finally, the integration of artificial intelligence techniques (AI) into medical informatics has raised significant concerns regarding the security and privacy concerns. Yoon, Stanley [18] quantified the privacy vulnerability of the MT-CNN model and proposed to apply vocabulary selection methods to alleviate privacy vulnerability while maintaining the same level of clinical task performance.

Overall, we observe that the existing studies presented in Table 1 have predominately focused on the extraction of a restricted set of coding item types, usually not exceeding five, exclusively from pathology reports. These investigations have largely overlooked the presence of diverse medical report types within a patient’s medical history. Neglecting these additional report types, which may contain valuable information or introduce complexities into the extraction process, raises questions about the practical utility of these models in real-world contexts, particularly in assisting registrars with their day-to-day responsibilities. There is the need for a comprehensive approach to ensure the broader applicability of automated extraction models.

### 1.2 Goal of this study

The objective of this study is to develop a cancer registry coding system that can effectively support registrars in their daily abstraction tasks. To accomplish this, we specifically focus on lung cancer as our research target and formulate the abstract process as to develop a system $F_{\theta }$ capable of processing all available reports R from a patient’s entire cancer treatment journey. This involves analyzing various types of unstructured electronic textual reports collected over a period of approximately 1.5 years to generate suggested coding results C, encompassing 30 coding types as defined in Table 2.

(1) $$F_{\theta }\left(R\right)=\{c_{i}|c_{i}\in C,\mathrm{where}\left|C\right|=30\}$$

**Table 2:** The definition of the 30 lung cancer coding items considered in this study.

Coding Type	Description
AJCC Edition	The version and chapters of the AJCC (American Joint Committee on Cancer) cancer staging manual used to determine the cancer stage of the case.
Behavior Code	The morphological code (M-code) in the pathological diagnosis. The 5th code in the M-code is the behavior code. The first four digits of M-code indicate the specific histological term. The fifth digit is the behavior code, which indicates whether a tumor is malignant, benign, in situ, or uncertain.
Clinical Other Staging Group	The classification standards of the selected “Other Staging Systems” (defined below) chosen for staging cancer cases
Clinical Stage Descriptor	The prefix or suffix used in conjunction with clinical TNM fields. The prefix/suffix denotes special circumstances that may affect the staging and analysis of the data and is based on the clinical T, N, and M categories prior to treatment.
Date of First Microscopic Confirmation	The earliest date when the case's cancer was confirmed by microscopy.
Date of First Surgical Procedure	The earliest date of surgery for cancer performed at any medical institution.
Date of Initial Diagnosis	The earliest date the cancer was diagnosed by a physician.
Date of Surgical Diagnostic and Staging Procedure	The date of the surgical treatment performed for diagnosis or staging at any medical institution.
Diagnostic Confirmation	The most accurate basis of diagnosis at the reporting hospital or an external hospital for the case.
Grade Clinical	The grading/differentiation of the solid tumor before the first treatment. Grading/differentiation refers to the degree of similarity between the tumor and normal tissues. Well differentiated (Grade I) is most similar to normal tissue; undifferentiated (Grade IV) is most dissimilar from normal tissue.
Grade Pathological	The grading/differentiation of the solid tumor after surgery at the primary site. Grading/differentiation refers to the degree of similarity between the tumor and normal tissues. Well differentiated (Grade I) is most similar to normal tissue; undifferentiated (Grade IV) is most dissimilar from normal tissue.
Histology	The structure of the primary tumor cells under the microscope.
Laterality	The specification of whether the cancer originates from one side of a pair of organs or the body. It is a only applicable to the primary tumor site.
Lymph vessels or Vascular Invasion	The code is recorded based on the pathological report of the primary site to indicate the presence or absence of invasion into lymph vessels or blood vessels.
Nodes Examined	The total number of regional lymph nodes examined by a pathologist.
Nodes Positive	The total number of positive regional lymph nodes examined by a pathologist.
Other Staging System	The selection of alternative staging criteria if the AJCC Cancer Staging System is not utilized.
Pathologic M	The presence of distant metastases of the primary tumor
Pathologic N	The regional lymph nodes involvement of the tumor. The item is encoded based on all clinical evaluations done prior to definitive surgery, plus all information through completion of definitive surgeries in the first course of treatment in the absence of disease progression or within 4 months of diagnosis, whichever is longer.
Pathologic Stage Descriptor	The prefix or suffix used in conjunction with pathologic TNM fields. The prefix/suffix denotes special circumstances that may affect the staging and analysis of the data and is based on the pathologic T, N, and M categories after completion of surgical treatment.
Pathologic T	The size of the primary tumor and its invasion into adjacent tissues. The item is encoded based on all clinical evaluations done prior to definitive surgery, plus all information through completion of definitive surgeries in the first course of treatment in the absence of disease progression or within 4 months of diagnosis, whichever is longer.
Perineural Invasion	The presence of neural invasion as noted in the pathological report of the primary site in the medical records.
Primary Site	The primary site of the cancer.
Scope of Regional Lymph Node Surgery	The extent of regional lymph nodes removed, sectioned, or aspirated during the primary site surgery or another separate surgery at the reporting hospital.
SSF 2	Cancer site-specific factors (SSF) related to prognosis and treatment decisions. SSF2: Visceral pleural Invasion (VPI)/elastic layer value set SSF5: Sampling or dissection of mediastinal lymph nodes (N2 Nodes) value set SSF6: EGFR (epidermal growth factor receptor ) gene mutation value set SSF7: ALK (Anaplastic lymphoma kinase) gene translocation value set
SSF 5	
SSF 6	
SSF 7	
Surgical Margins	The final status of the surgical margins after the primary tumor is removed.
Surgical Margins Distance	The closest distance of tumor cells to the surgical margins in the pathological report after the primary tumor is removed.

We assess the feasibility of directly employing state-of-the-art deep learning model architectures with learnable parameters θ, such as HAN and MT-CNN as presented in the previous subsection, for the task according to our formulation. We have also compared their performance with the proposed method with θ as the weighted rules outlined in Section 2. Additionally, we have elaborated on the integration of the developed system within a single hospital environment to demonstrate the practical applicability of our proposed approach in Section 3.

## 2 Materials and methods

### 2.1 Experimental data source and curation methods

According to the guidelines of the Declaration of Helsinki, the Institutional Review Board (IRB) of KMUH (KMUH-IRB-E(I)− 20210282) approved this study. The lung cancer patients were first identified from cancer registry data using the diagnostic code for lung cancer (International Classification of Diseases for Oncology, ICD-O-3: C33, C34) between January 1, 2018 and December 31, 2020. For these patients, we used the unique and encrypted identifiers to link their corresponding unstructured EHRs, including pathology and image reports such as computed tomography reports and magnetic resonance imaging reports. The entire EHRs also include medication and laboratory records, and can be extracted by the same way as unstructured data. Since the target data elements are primarily for cancer diagnosis, and do not require information from medication and laboratory, they were not included in the compiled dataset. There were totally 80,071 de-identified reports from 1287 cancer patients (path reports: 7409, image reports: 72,662). On average, each patient has 14.6 reports associated with their medical history.

The development and evaluation of our system relies on the official lung cancer registry coding records, which were meticulously assigned by certified cancer registrars and serves as our gold standard reference. The TCRC defines a comprehensive list of 115 items in the Taiwan cancer registry coding manual long form, with 16 of these items deem irrelevant to lung cancer cases. Additionally, eight items pertain to personal information which are already presented in a structured format, rendering them unrequired for NLP extraction. Consequently, the remaining 91 items can be extracted by NLP. However, certain privacy considerations, primary stemming from discharge summaries, prevent us from accessing and retrieving some of these items. Therefore, during the development phase of the system presented in this study, we excluded these privacy-sensitive items, ultimately narrowing our target to 30 key data elements for our research.

To create a lung cancer registry concept recognition corpus, we followed the similar annotation procedure suggested in our previous work [19] for colorectal cancer. Referencing Table 3, we delineated 26 cancer registry-related concepts within the annotation guidelines, instructing annotators to mark these concepts if mentioned in the provided reports. The selection of these lung cancer-related concepts was guided by expert consultations and consensus discussions, which aimed to identify and enumerate the key concepts crucial for accurate lung cancer registry coding. The corpus annotation procedure was conducted in two steps. Initially, we randomly selected 103 patients along with their corresponding medical reports from the original lung cancer corpus. Subsequently, we engaged four annotators to perform annotations on the sampled reports adhering to a predefined annotation guideline. Throughout the annotation process, the annotators meticulously cross-referenced the annotations made within the reports with the gold standard results from the corresponding registry records. Furthermore, they provided suggestions for the formulation of rules to be integrated into our symbolic AI systems, as elaborated in Section 2.2.

**Table 3:** Annotation statistics for the lung CR concept recognition corpus.

Report Type	Pathology Report	Image Report
Report	2344	5034
Patient	507	507
Nodes Examined	1491	4173
Nodes Positive	1451	3139
Tumor Size	606	389
Histology	2818	1347
Grade	1045	736
Pathologic T	352	146
Pathologic N	303	135
Pathologic M	39	82
Clinical T	1	364
Clinical N	2	358
Clinical M	1	286
Clinical Stage Group	0	7
Behavior Code	1296	0
Primary Site	45	1720
Perineural Invasion	12	1
Lymph vessels or Vascular Invasion	9	2
Surgical Margins	7	0
Surgical Margins Distance	2	0
SSF1	0	24
SSF2	3	10
N2 Nodes	44	0
SSF6	11	0
SSF7	7	0
ICD10CM	1683	593
ICD_O_3_SITE	1590	2
Stage Classification	362	188

Following the completion of the annotation tasks, we evaluated the inter-annotator agreement to ascertain the degree of agreement among the annotators regarding the annotations made within the given reports. After achieving a strong level of agreement (the Kappa value > 0.8) [20], we progressed to the second stage, in which the remaining reports were evenly distributed among the four annotators. Their task was to identify and annotate specific text spans corresponding to a pre-defined list of clinical concepts essential for determining the lung cancer registry coding.

### 2.2 Development of the lung cancer registry coding system

In this study, we enhanced the hybrid neural symbolic system originally introduced in our prior research [12]. This enhancement involved the incorporation of weighted rules specifically designed for determining the target coding items from the various reports found within a patient’s medical history. Unlike the original system, which concentrated solely on colorectal cancer and its eight registry coding types, we significantly expanded the system's capabilities in this study to encompass lung cancer and accommodate a total of 30 distinct coding types.

The enhanced system contains three main components. The first is the preprocess module based on our clinical toolkit [21], [22], which was employed to segment sentences, generate the corresponding tokens, and recognize section headings, such as “Microscopic Examination”. The preprocessed sentences are then analyzed by the recognition and normalization module described in Section 2.2.1 to extract key clinical concepts. The concepts along with the preprocessed information are established as new facts in the fact database used by the expert system module introduced in the Section 2.2.2. Starting with the new and known facts in the fact database, the expert system applies the forward-chaining algorithm to trigger all weighted rules predefined within the knowledge base when their antecedents align with the current set of facts. Subsequently, the conclusions derived from these rules are integrated into the fact database, thereby establishing new pieces of information as known facts. This iterative process continues until no further rules can be triggered, signifying that all relevant rules have been applied and all potential coding results have been determined. Further elaboration on the enhancements made to the second and third modules will be provided in the subsequent subsections.

#### 2.2.1 Cancer registry coding fact extraction module

The primary objective of this module is to extract pertinent coding-related information from unstructured texts. To expedite our development process for the lung cancer registry coding task, we harnessed the power of transfer learning. Specifically, we leveraged our established colorectal cancer registry concept recognition model [12] as a pre-trained model. To adapt it for the specific requirements of this study, we modified the model by increasing the number of output nodes in its final multilayer perceptron layer to align with the number of target concept types defined for lung cancer registry tasks. Following this adjustment, we conducted a fine-tuning process using the dedicated corpus compiled in this study, aiming to optimize its performance in extracting clinical concepts relevant to the lung cancer registry.

Furthermore, concept mentions such as histology and primary site extracted by the above model were normalized by using an enhanced dictionary lookup approach [23]. For example, description like “lung, left upper lobe” is normalized to the unified medical language system (UMLS) [24] concept unique identifier (CUID) “C1261076″. Similarly, information such as surgery code for lung was also extracted through a dictionary-based method. These recognized cancer registry concepts were then converted into facts and stored within the fact database for the reasoning process of the developed symbolic AI system.

#### 2.2.2 Cancer registry coding generation

We interviewed the cancer registrars and followed the Taiwan cancer registry coding manual long form to encode the knowledge for lung cancer registry coding in our symbolic AI system. The developed system relies on the compiled rules and facts formed by the concepts mentioned above to infer the codes. The fact database in the system is first reset to the initial state before considering any new facts extracted from all available reports for a given patient journey. Next, we populate the fact database with the pertinent lung cancer registry-related data by leveraging identified cancer registry concepts. This involves incorporating details such as the associated report section header [21] and co-occurrence information of concepts within the same sentence. Finally, the inference engine uses all weighted rules to deduce the lung cancer registry results from the existing known facts in the fact database.

Each rule consists of the antecedent and the consequent parts. The antecedent of a rule represents the desired condition to be satisfied for firing the rule. A rule can have multiple antecedents joined by the keyword AND. In our implementation, an antecedent is represented by a fact object with the desired value linked by an operator. For example, the antecedent below represents a condition in which the UMLS CUID (concept unique identifier) of the recognized primary site/laterality is “C1261076″

C.type=′PrimarySiteLaterality′ANDC.UMLS=′C1261076′

The antecedent of a rule is matched against the observed facts stored in the fact database. On the other hand, the consequent of a rule indicates the action to be performed if the antecedent is satisfied. For instance, the antecedent in the following rule is satisfied if the concept is described under the “Diagnosis” section and co-occurs with histology concepts in the same sentence.

$$C.\mathrm{section}{=}^{\prime }{\mathrm{Diagnosis}}^{\prime }\mathrm{AND}C.\mathrm{cooccurHistology}=\mathrm{True}\to$$

$$D.\mathrm{type}=\mathrm{\prime PRIMARY\_SITE\_CODING\prime}\mathrm{AND}D.\mathrm{value}{=}^{\prime }C341^{\prime }\mathrm{AND}E.\mathrm{type}=\mathrm{\prime LATERALITY\_CODING\prime}\mathrm{AND}E.\mathrm{value}=\prime 2\prime$$

The subsequent action is then to update the database with new facts D whose ‘‘type’’ and ‘‘value’’ attributes are set to ‘‘PRIMARY_SITE_CODING’’ and “C341″, respectively, and E whose ‘‘type’’ and ‘‘value’’ attributes are set to ‘‘LATERALITY_CODING’’ and “2”, respectively.

During the process, new facts are inferred and added to the fact database when the antecedences associated with rules satisfy the known facts. This process will iterate until no new inferences are possible, and the final coding result is obtained and ranked according to the associated weights. The final code assigned to the item was the code of the coding item with the highest weight.

#### 2.2.3 Weight estimation

**Fig. 1:**
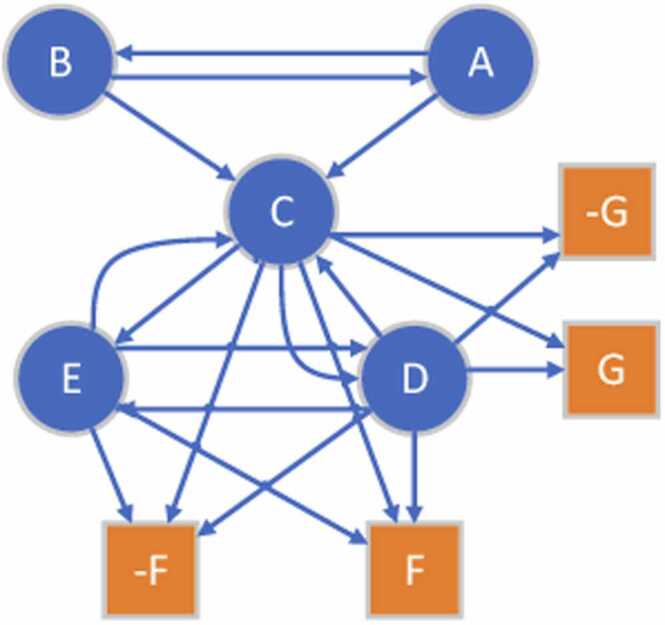
An example graph skeleton created for the sample rule set: $\left\{\begin{array}{c} A\mathrm{AND}B⟶C\\ C\mathrm{AND}D\mathrm{AND}\;E⟶F\\ C\mathrm{AND}D⟶G \end{array}\right\}$.

To estimate the weight associated with each rule, we propose a graph-based approach which considers the co-occurrence frequencies of the antecedent and the consequent parts. A directed and weighted graph G is used to represent the subgraph patterns of the defined rules for a CR coding type c∈C. In this graph, each vertex $v_{i}$ of G is an antecedent or consequence in a rule. We use the notation R to denote the set of all antecedents and consequences. To describe the composition of two antecedents, i.e*.*, antecedent $v_{i}$ and $v_{j}$, the graph skeleton is created as follows. For each antecedent pair $\left(v_{i},v_{j}\right)$, if $v_{i}$ and $v_{j}$ occur in a rule R of a coding type c, we create two edges $\langle i,j\rangle$ and $\langle j,i\rangle$ to G. For each antecedent-consequence pair $\left(v_{i},v_{k}\right)$, if $v_{i}$ and $v_{k}$ occur in a rule R of a coding type c, we instead create one more vertex, $v_{k^{\prime }}$, and add two edges, one from i to k and the other from i to $k^{\prime }$, to G, where $v_{k}$ and $v_{k^{\prime }}$ represent the assertions for the coding type c are true or false, respectively. Fig. 1 illustrates an example graph constructed for the following rule set defined for coding F and G:

$$\left\{\begin{array}{c} A\mathrm{AND}B⟶C\\ C\mathrm{AND}D\mathrm{AND}\;E⟶F\\ C\mathrm{AND}D⟶G \end{array}\right\}$$

Here, we create a directed graph because such antecedent/consequence relationships are asymmetric in nature, since two antecedents may not have the same influence either on each other or the consequences in a rule. One antecedent $v_{i}$ could be a major factor of a consequence but not for all. Therefore, we use directed and weighted edges to describe such dual relationships between antecedents and consequences by quantifying such influence in an adjacency matrix $Α\in R^{\left(\left|R\right|+\left|\mathrm{Con}\right|)\times (\left|R\right|+\left|\mathrm{Con}\right|\right)}$, where $\left|\mathrm{Con}\right|$ refers to the number of consequences and each element ${Α}_{\mathrm{ij}}$ represents the weight of edge $\langle i,j\rangle$. Note that there is only one edge, $\langle i,j\rangle$, present connecting the antecedent vertex ($v_{i}$) to the consequence vertex ($v_{j}$) defined in a rule. In addition, it is possible that the fired consequences don’t match with the registry codes assigned by the registrars, therefore the non-match consequence are added as illustrated in Fig. 1 denoted by prefixing the ‘-’ symbol.

To model the influence of antecedents, we first define a co-occurrence matrix, $Β\in N^{\left(\left|R\right|+\left|\mathrm{Con}\right|)\times (\left|R\right|+\left|\mathrm{Con}\right|\right)}$, initialized with all zeros. When calculating the values of Β, we increase the elements ${Β}_{\mathrm{ij}}$ and ${Β}_{\mathrm{ji}}$, which represent the antecedents, by 1 for each co-occurrence pair of $v_{i}$ and $v_{j}$ when the two antecedents are matched with the observed facts when processing each record of all patients. When a rule is fired and the consequences leading to the generation of cancer registry codes, we check whether the consequences are matched with the codes assigned by registrars; if it is matched, ${Β}_{\mathrm{ij}}$, which represents the antecedent-consequence pair $\left(v_{i},v_{k}\right)$, is increased by 1, otherwise ${Β}_{ij^{\prime }}$, where $j^{\prime }$ is the index of the non-match consequence corresponding to the consequence j, is increased by 1. Let $q_{i}=\sum_{j=1}^{\left|\left|R\right|+\left|\mathrm{Con}\right|)\right|}{Β}_{\mathrm{ij}}$ be the sum of the i-th row of B, then, the weighted adjacency matrix Α is calculated as follows:

$${Α}_{\mathrm{ij}}=\left\{\begin{array}{cc} 0 & \mathrm{if}i=j\mathrm{and}q_{i}\neq 0\\ 1 & \mathrm{if}i=j\mathrm{and}q_{i}=0\\ \frac{{Β}_{\mathrm{ij}}}{q_{i}} & \mathrm{otherwise} \end{array}\right)$$

Α is typically not symmetric, which makes G a weighted and directed graph. The element ${Α}_{\mathrm{ij}}$ measures the frequency of the antecedent/consequence pair ($v_{i},v_{j}$) in all co-occurrence pairs of $v_{i}$. A higher frequency implies $v_{j}$ appears more times along with $v_{i}$ than others. Therefore, we can infer $v_{j}$ has more influence on $v_{i}$.

Based on the estimated weighted adjacency matrix, we then estimate the deterministic weight for a rule with the following message-passing mechanism using the compiled training set. During each message-passing phase in the created G, the weight $w_{i}$ corresponding to the node $v_{i}$ is updated according to the frequency information aggregated from $v_{i}$’s neighborhood $N(v_{i})$ who themselves are informed by their own neighbors. The message-passing update can then be expressed as follows: $w_{i}=\sum_{j\in N(v_{i})}{Α}_{\mathrm{ji}}$. In Fig. 1, consider the target consequence F, intuitively, F gets all the messages from its neighborhood nodes {C,D,E} and the nodes {C,D,E} in turn processes information from their neighbors. For example, D aggregates information from {C, E}, while C and A aggregate information from {A,B,D,E} and {B} respectively.

### 2.3 Performance evaluation method

To develop the hybrid neural symbolic system, we split the de-identified reports of patients into the ratio of 80:20 for compiling the training and test sets. Then, the developed system's performance was evaluated on the test set using three metrics: precision (P), recall (R), and F_β_-measure (F_β_), which were commonly used for evaluating the performance of NLP systems [25], [26], [27]. The formulae for the three metrics are defined as follows:

(2) $$P=\frac{\mathrm{TP}}{\mathrm{TP}+\mathrm{FP}}$$

(3) $$R=\frac{\mathrm{TP}}{\mathrm{TP}+FN}$$

(4) $$F_{\beta }=(1+\beta^{2})\times \frac{P\times R}{\left(\beta^{2}\times P\right)+R}$$

In the above formulae, TP, FP, and FN represent the number of true positive (TP), false positive (FP), and false negative (FN) results for each registry item type, respectively. Precision and recall are also known as positive predictive value and sensitivity. The F_β_ is the weighted harmonic mean of the P and R. In this work, the value of β = 1 is used and the F_β_-measure is in micro averaging.

### 2.4 System interface and integration

**Fig. 2:**
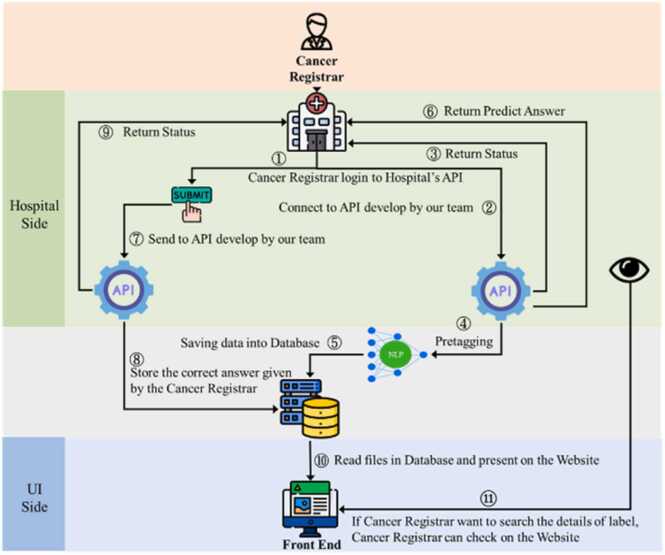
Flowchart of the developed system working within the hospital information system environment.

To integrate the developed system within the regular workflow of the cancer registrars in hospitals, we delivered the system as a web service to KMUH, which provides an application programming interface (API) accepting the reports of a patient to infer the codes for the target coding items. Fig. 2 depicts the integrated workflow flowchart.

When an authorized cancer registrar begins coding by requesting reports from the hospital information system (HIS) for a target patient (Step 1), these reports are also forwarded to our developed system via the web service API (Steps 2 and 3). After recognizing the clinical concepts and inferring their codes (Step 4), the predicted outputs are stored in our database (Step 5) and sent back to the user interface used by the cancer registrar (Step 6) to aid the coding work. Finally, after confirming the predicted registry items in the user interface (Step 7), the confirmed results are stored in both the original database in the HIS environment and our database (Step 8). Additionally, cancer registrars can examine the details of the inference steps to understand how the system deduces the results of a CR item by clicking on the eye icon available on the user interface (Steps 10 and 11).

## 3 Results and discussion

### 3.1 Corpus statistics

A total of 7378 reports got annotated from the 507 accounts sampling of lung cancer patients in the original corpus. Table 3 illustrates a statistical summary of the annotated results of the lung cancer registry concept recognition corpus. The final Kappa value k is 0.842 (Almost Perfect) [28].

After analyzing the collected data, we discovered that the collected cancer registry records contained cancers other than lung cancer, so we excluded registry records that matched the following two cases: 1) records that are not related to lung cancer were removed through the primary site information in the cancer registry records and 2) patients with only a few reports (<2) or with only one type of report. The final test set used in this study contains 342 patients’ lung cancer registry records.

### 3.2 Experimental results

To illustrate the effectiveness of the developed system, we implemented three baselines for comparison. The first is the mode configuration in which, for a given lung cancer registry item, the most frequently occurring label that appeared in the training data is output for a patient. The second and third baselines are the HAN and MT-CNN models introduced in the “Priori Work” section. For HAN, we followed the binary relevance transformation method [29] to formulate the coding task for each CR item as a multiclass classification task to train the corresponding numbers of HAN-based classifiers. For MT-CNN, BioWordVec [30] were used to represent word tokens and only one model was trained for generating all of the 30 cancer registry items. Table 4 shows the coding performance on the test set of all developed systems. In general, both supervised learning approaches, including HAN, MT-CNN and the proposed hybrid neural symbolic system, apparently outperformed the mode configuration in almost all coding items. HAN outperformed MT-CNN among more registry items while the hybrid neural symbolic system achieved better F-scores than HAN in 23 out of 30 items. Please note that as both the mode configuration and the HAN and MT-CNN models generate outputs for all tested patients’ records, the assessment of their F-scores is equivalent to the evaluation of their PR-scores and accuracy.

**Table 4:** Performance comparison among the 30 lung CR items of the developed systems. The best PRF-scores per type are represented in bold.

**Lung CR Item Type**	**Mode**	**Neural Symbolic System**			**HAN**	**MT-CNN**
	F*	P	R	F	F*	F*
AJCC Edition	0.941	**1.000**	**1.000**	**1.000**	0.913	0.941
Date of First Surgical Procedure	0.466	**0.978**	**0.975**	**0.976**	0.525	0.969
Diagnostic Confirmation	**0.971**	**0.971**	**0.971**	**0.971**	0.946	0.969
Date of Initial Diagnosis	0.011	**0.975**	**0.940**	**0.957**	0.000	0.000
Other Staging System	0.928	**0.956**	**0.956**	**0.956**	0.942	0.928
Clinical Other Staging Group	0.928	**0.956**	**0.956**	**0.956**	0.942	0.928
SSF2	0.547	**0.945**	**0.952**	**0.956**	0.862	0.790
Laterality	0.547	**0.953**	0.942	0.948	**0.951**	0.910
Date of First Microscopic Confirmation	0.005	**0.982**	**0.914**	**0.947**	0.004	0.004
Pathologic Stage Descriptor	0.941	**0.947**	**0.945**	**0.946**	0.936	0.910
SSF7	0.448	**0.942**	**0.939**	**0.940**	0.753	0.690
Surgical Margins	0.520	**0.939**	**0.939**	**0.939**	0.829	0.750
Behavior Code	0.971	**0.990**	0.884	0.934	**0.994**	**0.994**
Grade Pathological	0.566	**0.944**	0.920	0.932	**0.939**	0.797
Pathologic M	0.799	**0.930**	**0.930**	**0.930**	0.822	0.799
Pathologic N	0.534	**0.930**	**0.927**	**0.928**	0.904	0.830
Scope of Regional Lymph Node Surgery	0.517	**0.915**	**0.913**	**0.914**	0.832	0.750
Pathologic T	0.525	**0.915**	**0.894**	**0.905**	0.763	0.730
Clinical Stage Descriptor	0.702	**0.904**	**0.896**	**0.900**	0.738	0.820
Grade Clinical	0.576	**0.901**	0.893	0.897	**0.900**	0.742
Nodes Positive	0.515	**0.895**	**0.892**	**0.893**	0.862	0.810
Primary Site	0.531	**0.892**	**0.876**	**0.884**	0.710	0.750
Nodes Examined	0.515	**0.880**	**0.875**	**0.878**	0.658	0.550
Perineural Invasion	0.418	**0.880**	0.867	0.874	**0.878**	0.814
Histology	0.375	**0.885**	**0.857**	**0.871**	0.760	0.700
Date of Surgical Diagnostic and Staging Procedure	0.346	**0.870**	**0.861**	**0.866**	0.325	0.304
Lymph vessels or Vascular Invasion	0.410	**0.871**	0.859	0.865	**0.868**	0.797
SSF6	0.531	**0.865**	**0.856**	**0.860**	0.526	0.526
Surgical Margins Distance	0.520	**0.868**	0.848	0.858	**0.864**	0.533
SSF5	0.550	**0.863**	**0.848**	**0.856**	0.754	0.600

* Because for each cancer registry item of a given patient, the baseline, HAN and MTCNN will generate one corresponding output, the value of p/r/f will have the same value.

The mode configuration can have F-scores greater than 0.9 on some coding items, such as "Behavior Code", "AJCC Edition", "Other Staging System’, "Diagnostic Confirmation", and so on, indicating skewed label distributions for those items. Furthermore, we can see that the neural symbolic system achieves higher precision than the other two supervised learning methods. We can also observe that these two models perform worse on items that involve numerical values, such as "Nodes Examined" and items associated with temporal information. The fact that the multiclass classification formulation may be inappropriate for these item types is one of the primary causes of their poor performance in those items.

#### 3.2.1 The effectiveness of weighted rules

In Table 5, we study the effectiveness of the incorporating the weighted rules in our neural symbolic system by conducting the ablation study. We observed that for certain coding items like site-specific factors (SSFs), “Behavior” and “Pathologic Stage Descriptor”, the inclusion of the weighted rules doesn’t yield any performance improvement. This indicates that the supporting evidences for these items might be present in only a single report among all of the patient’s medical records. However, for most of the items, ignoring the weights assigned for the rules can significantly reduce both the PR-scores resulting in lower F-scores. For example, the F-scores of “Primary Site” and “Laterality” drop from 0.884 and 0.948 to 0.286 and 0.374, respectively. In contrast to the colorectal cancer registry task investigated in our previous study [12], the task setting in this study closely mirrors the real hospital setting, wherein all encountered during a patient’s journey are gathered for analysis. Moreover, considering that lung cancer patients may receive diagnoses of multiple primary lung cancers, the inclusion of weighted rules becomes necessary. However, it is essential to note that the weights’ effect may vary for different target cancers and datasets.

**Table 5:** Comparative performance of systems using weighted and unweighted rules.

**Item Type**	**Unweighted Rules**			**Weighted Rules**		
	**P**	**R**	**F**	**P**	**R**	**F**
AJCC Edition	1.000	1.000	1.000	1.000	1.000	1.000
Date of First Surgical Procedure	0.978	0.975	0.976	0.978	0.975	0.976
Diagnostic Confirmation	0.971	0.971	0.971	0.971	0.971	0.971
Date of Initial Diagnosis	0.944	0.785	0.857	**0.975**	**0.940**	**0.957**
Other Staging System	0.956	0.956	0.956	0.956	0.956	0.956
Clinical Other Staging Group	0.956	0.956	0.956	0.956	0.956	0.956
SSF2	0.959	0.945	0.952	0.959	0.945	0.952
Laterality	0.377	0.371	0.374	**0.953**	**0.942**	**0.948**
Date of First Microscopic Confirmation	0.840	0.755	0.795	**0.982**	**0.914**	**0.947**
Pathologic Stage Descriptor	0.947	0.945	0.946	0.947	0.945	0.946
SSF7	0.942	0.939	0.940	0.942	0.939	0.940
Surgical Margins	0.561	0.547	0.554	**0.939**	**0.939**	**0.939**
Behavior Code	0.993	0.884	0.934	0.990	0.884	0.934
Grade Pathological	0.591	0.575	0.583	**0.944**	**0.920**	**0.932**
Pathologic M	0.787	0.787	0.787	**0.930**	**0.930**	**0.930**
Pathologic N	0.930	0.927	0.929	0.930	0.927	0.928
Scope of Regional Lymph Node Surgery	0.535	0.523	0.529	**0.915**	**0.913**	**0.914**
Pathologic T	0.573	0.558	0.566	**0.915**	**0.894**	**0.905**
Clinical Stage Descriptor	0.696	0.690	0.693	**0.904**	**0.896**	**0.900**
Grade Clinical	0.579	0.577	0.578	**0.901**	**0.893**	**0.897**
Nodes Positive	0.889	0.886	0.888	**0.895**	**0.892**	**0.893**
Primary Site	0.290	0.283	0.286	**0.892**	**0.876**	**0.884**
Nodes Examined	0.529	0.517	0.523	**0.880**	**0.875**	**0.878**
Perineural Invasion	0.874	0.862	0.868	**0.880**	**0.867**	**0.874**
Histology	0.608	0.589	0.598	**0.885**	**0.857**	**0.871**
Date of Surgical Diagnostic and Staging Procedure	0.843	0.832	0.837	**0.870**	**0.861**	**0.866**
Lymph vessels or Vascular Invasion	0.868	0.856	0.862	**0.871**	**0.859**	**0.865**
SSF6	0.865	0.856	0.860	0.865	0.856	0.860
Surgical Margins Distance	0.561	0.547	0.554	**0.868**	**0.848**	**0.858**
SSF5	0.863	0.848	0.856	0.863	0.848	0.856

#### 3.2.2 Correlation analysis: report types and coding items

Table 1 showcases a notable departure from existing studies, which predominantly concentrate on extracting a limited range of coding item types solely from pathology reports. In contrast, our dataset encompasses 30 coding items, reflecting a diverse type of reports associated with each patient. These reports can be broadly categorized into two primary types: pathology and image reports. Consequently, numerous compiled rules within our system incorporate information about the report types in their antecedent parts to prioritize candidate codes based on the type of report. Interestingly, we observed consistent patterns in the associated weights for different coding-report type pairs, prompting us to employ backward-chaining of our symbolic expert system to investigate the correlation between report types and the origin of coding items. The analysis unveiled a strong association between registry items and the specific report type as summarized in Table 6. These findings offering insights and recommendations for enhancing the training process of end-to-end learning algorithms such as HAN and MT-CNN implemented in this study through the incorporation of report types into their models. We anticipate that integrating representations of report types into these models in future research will greatly enhance their performance and practical utility in supporting the abstraction process within hospital environments.

**Table 6:** Model training dataset column description.

**Report Type**	**Lung Cancer Registry Item Type**		
**Pathology Report**	Grade Pathological	AJCC Edition	Behavior Code
	Surgical Margins	Nodes Examined	SSF2
	Pathologic T	SSF7	Pathologic Stage Descriptor
	Histology	Clinical Other Staging Group	Lymph vessels or Vascular Invasion
	Scope of Regional Lymph Node Surgery	Pathologic M	SSF6
	Other Staging System	Grade Clinical	Surgical Margins Distance
	Perineural Invasion	SSF5	Nodes Positive
	Diagnostic Confirmation	Pathologic N	Date of Surgical Diagnostic and Staging Procedure
	Date of First Microscopic Confirmation		
**Imaging Report**	Primary Site	Laterality	Clinical Stage Descriptor
	Date of Initial Diagnosis	Date of First Surgical Procedure	

### 3.3 Real hospital environment results

#### 3.3.1 Front-end user interface of the developed lung cancer registry coding system

**Fig. 3:**
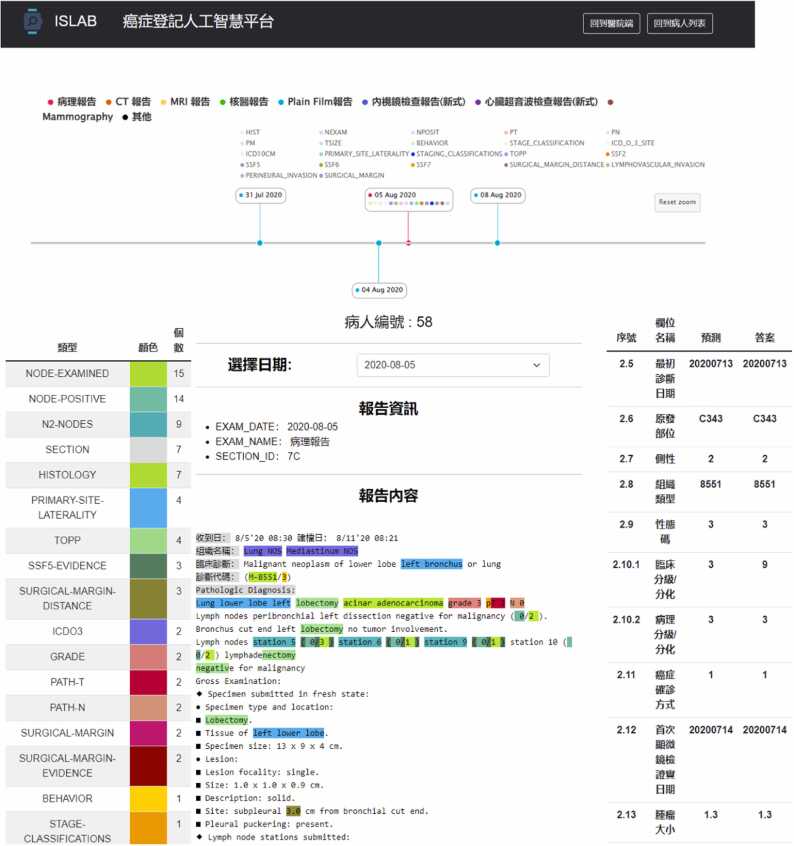
The front-end of the developed lung cancer registry coding visualization system for a patient journey visualization platform system for the integration of the KMUH HIS.

Fig. 3 demonstrates the front-end interface developed for integration in a hospital environment. The front end provides various information for the cancer registrar, including the timeline of the reports for a target patient and the report content highlighted with the important cancer registry concepts automatically recognized by our system. This user interface aligns with Step 11 depicted in Fig. 2, where the registrar can utilize the interface to navigate through all reports of a patient annotated with significant registry items. It enables them to investigate the rationale behind the system’s prediction for a particular registry item.

**Fig. 4:**
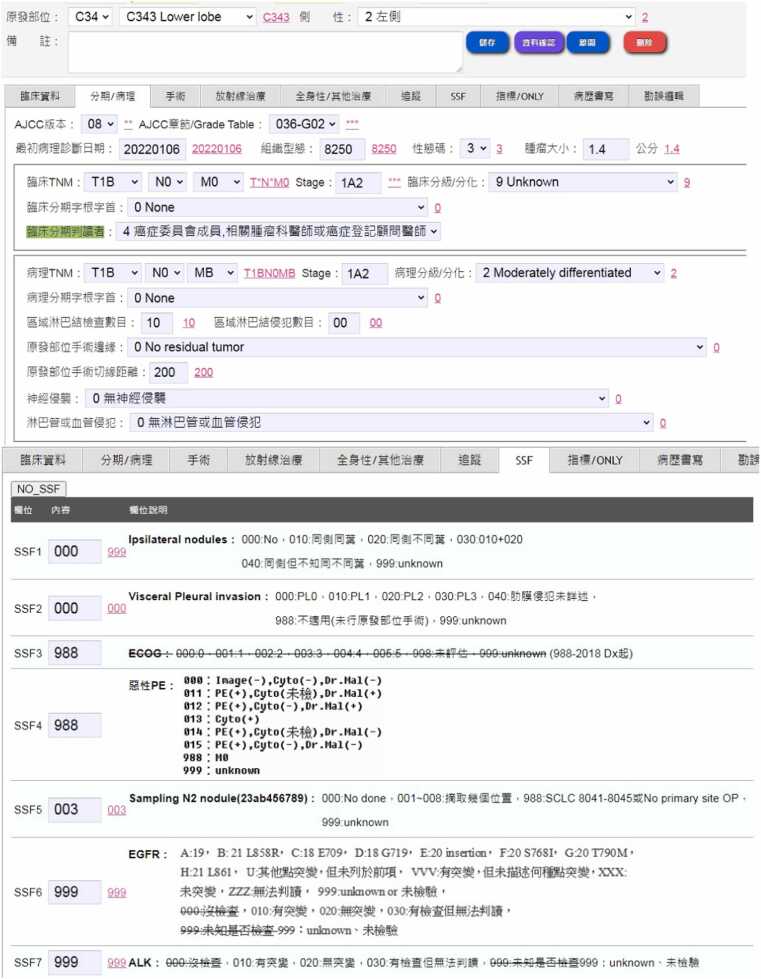
The enhanced cancer registry coding user interface, integrated into the KMUH HIS and used by KMUH registrars. The recommended coding results are highlighted in red behind each respective input field.

The cancer registry user interface, routinely used by cancer registrars in KMUH, is illustrated in Fig. 4. The interface has been enhanced to integrate coding results predicted by our system. Currently, the typical usage scenario for presenting coding suggestions in the hospital’s coding process o is as follow: after completing their abstraction process, registrars click the “資料確認” (Data Check) button (depicted as the purple button in the upper subfigure of Fig. 4). The coding results recommended by our system in Step 6 (as depicted in Fig. 2) are then displayed alongside each input field. Registrars can review and cross-reference their coding outcomes with the suggested ones to mitigate coding errors. Therefore, in the current setting, the integrated system serves as an auditing tool [31], drawing attention to potential coding errors for registrars. Moving forward, we plan to further enhance user functionality by incorporating backward-chaining query support into the user interface provided in Fig. 3, empowering registrars to inquire about the rationale behind the coding output generated by the developed system, adding a layer of transparency and aiding in the continuous improvement of the abstraction process.

### 3.4 Estimation of the system performance in the real hospital environment

**Fig. 5:**
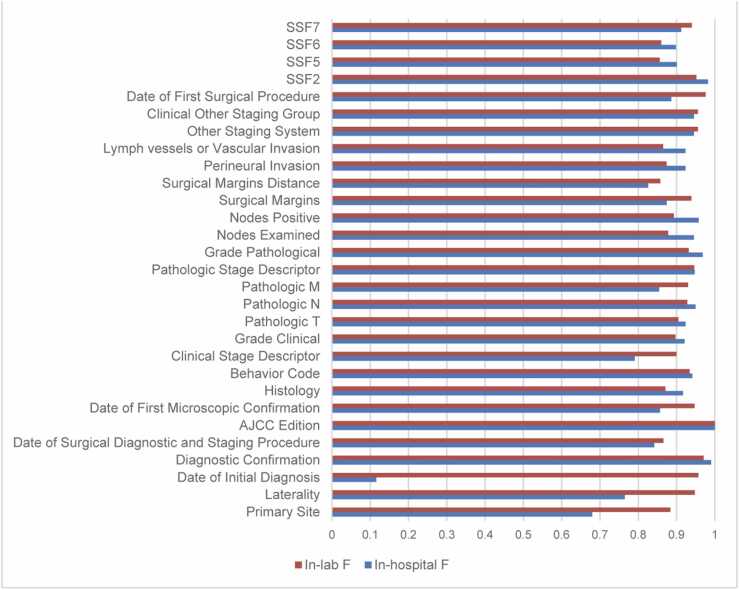
System performance in the real hospital environment. The red bar shows the F-score of the target registry item evaluated on the hold-out test set, while the blue bar illustrates the corresponding F-score estimated based on registrars’ abstraction results in the real hospital setting.

Fig. 5 shows the performance of the developed system integrated into the routine abstraction workflow of KMUH registrars, following the flowchart shown in Fig. 2. The experiment spanned from September 2022 to March 2023, during which our system processed reports from 260 lung cancer patients. The system’s outputs were then compared with the abstraction results manually recorded by the registrars.

As depicted in the figure, 25 out of 30 items exhibit F-scores exceeding 0.8. Notably, the deployed system demonstrated superior F-scores in the real environment, particularly for items such as “Diagnostic Confirmation”, “Histology”, “Behavior Code”, “Grade Clinical”, “Pathologic T”, “Pathologic N”, “Pathologic Stage Descriptor”, “Grade Pathological”, “Node Examined”, “Node Positive”, “Perineural Invasion”, “Lymph Vessels or Vascular Invasion”, “SSF2″, “SSF5″, and “SSF6″. While the “Date of Initial Diagnosis” item displays a notably low F-score in Fig. 5, we attribute this result to potential challenges in the data flow integration. Upon re-executing our system on the IRB-approved dataset, consisting of the same 260 patients’ reports involved in this experiment, independent of the integrated workflow in the hospital setting, we observed that the system achieved nearly the same F-score as the in-laboratory results depicted in Fig. 5. This suggests that the lower F-score observed in the integrated workflow may be linked to issues in data integration rather than inherent limitations of the system's performance. The outcomes conclusively affirm the resilience and effectiveness of the proposed system.

### 3.5 Limitations

This study acknowledges several limitations to ensure a nuanced interpretation of its findings. Firstly, the reliance on a lung cancer dataset obtained from a single medical center introduces a constraint on the study's generalizability. The limited scope of the dataset raises questions about the broader applicability of the developed system to diverse hospitals or regions. Thus, it is imperative to validate the system's performance on a more varied and extensive dataset that encompasses multiple medical centers.

Moreover, while the system demonstrated efficacy in lung cancer registry coding, its generalizability to other cancer types remains uncertain. The specific focus on lung cancer may constrain the system's adaptability to different cancer categories, underscoring the need for comprehensive evaluations across various cancer types. Future research endeavors should explore the system's performance in diverse healthcare settings and with a broader range of cancer datasets to enhance its robustness and applicability across different contexts.

## 4 Conclusion

In conclusion, our study presents a significant advancement in cancer registry practices through the integration of predictive coding and a user-centric interface. The primary challenge of the cancer registry coding task lies in the meticulous consideration of diverse and unstructured free-text reports, which, results in a time-consuming and labor-intensive abstraction process with the increasing the complexity of the coding rules. Our work addresses these challenges by enhancing a hybrid neural symbolic system with weighted rules specifically tailored for lung cancer registry coding using free-text reports, encompassing pathology and imaging reports. The developed system achieves commendable F-scores exceeding 0.85 across 30 coding items, showcasing its effectiveness in navigating the intricacies of the coding abstraction process.

Furthermore, the deployment of the system within the actual hospital environment demonstrated robust performance, particularly excelling in registry items such as “Diagnostic Confirmation,” “Histology,” and “Pathologic Staging.” The integration within the HIS in the cooperated hospital now provides cancer registrars with a patient journey visualization platform, significantly reducing coding errors and enhancing overall efficiency of the abstraction work. Feedback from registrars highlights the system’s reliability as a tool for assisting and auditing the abstraction process. By presenting key registry items along the timeline of a patient’s reports and providing accurate code predictions, the system improves the quality of registrar outcomes and reduces the labor resources and time required for data abstraction. In sum, this study underscores the potential of NLP, deep learning and symbolic AI techniques in elevating the accuracy and efficiency of cancer registry coding processes. The developed system holds promise for facilitating data collection and analysis in cancer research, as well as contributing to improve patient care and outcomes.

## Funding

This work was funded by the Health Promotion Administration, 10.13039/100008903Ministry of Health and Welfare (A1100302) and National Science and Technology Council [NSTC 112–2221-E-992−056 -MY3].

## CRediT authorship contribution statement

**Wen-Tsung Tsai:** Resources. **Ming-Yii Huang:** Project administration. **Chih-Jen Huang:** Project administration. **Chih-Jen Yang:** Project administration. **Chi-Yen Huang:** Conceptualization, Resources. **Hong-Jie Dai:** Conceptualization, Formal analysis, Funding acquisition, Investigation, Methodology, Project administration, Resources, Software, Supervision, Validation, Visualization, Writing – original draft, Writing – review & editing. **William Yu Chung Wang:** Writing – review & editing. **Chien-Chang Chen:** Resources, Writing – review & editing. **Inn-Wen Chong:** Conceptualization, Resources. **Tatheer Hussain:** Writing – original draft, Writing – review & editing. **Yi-Hsin Yang:** Conceptualization, Funding acquisition, Project administration, Supervision, Writing – review & editing. **Ting-Yu Wang:** Data curation, Formal analysis, Project administration, Software, Writing – original draft, Writing – review & editing. **Chen-Kai Wang:** Methodology, Software. **Ya-Chen Chang:** Project administration, Resources. **Su-Jung Yu:** Data curation, Validation. **Yi-Wen Shen:** Data curation, Validation. **Chen-Jiun Huang:** Software. **Chia-Hsuan Tsai:** Data curation. **Ching-Yun Wang:** Data curation. **Hsiao-Jou Chen:** Data curation. **Pei-Shan Weng:** Data curation. **You-Xiang Lin:** Software. **Sheng-Wei Chen:** Software. **Ming-Ju Tsai:** Project administration, Resources. **Shian-Fei Juang:** Resources. **Su-Ying Wu:** Resources. **Ping-Zun Liu:** Conceptualization, Resources.

## Declaration of Competing Interest

The authors declare no conflict of interest.
